# Whole-genome sequencing-driven source tracing of foodborne diseases: from cgMLST/SNP typing toward an integrated framework of pan-genomics and machine learning

**DOI:** 10.3389/fpubh.2026.1968969

**Published:** 2026-10-20

**Authors:** Yong Song, Chi Zhang, Jing-jing Li, Shi-yu Dai, Qin Zhang, Zhen-zhen Yao, Ying-Ying Ouyang, Chen-rui Gong

**Affiliations:** Health Monitoring Testing and Protection Institute, Hubei Provincial Center for Disease Control and Prevention, Wuhan, Hubei, China

**Keywords:** cgMLST, explainable artificial intelligence, foodborne disease, machine learning, pan-genome, source tracing, whole-genome sequencing

## Abstract

Core-genome multilocus sequence typing (cgMLST) and core-genome single-nucleotide polymorphism (cgSNP) analysis have made whole-genome sequencing (WGS) the standard tool for foodborne disease surveillance. Fixed-threshold clustering is increasingly unreliable for highly clonal lineages such as *Salmonella Enteritidis* ST11 and *Escherichia coli* ST131, whose core genomes differ by only a handful of alleles even between unrelated strains. The central question is whether surveillance should keep simple, easy-to-interpret allele thresholds or move to probabilistic machine-learning models that also use accessory-genome variation. Accessory genes and mobile genetic elements, including prophages, plasmids, and genomic islands, change on timescales that match outbreak transmission and carry niche-adaptation signal absent from the core genome. Gradient-boosting and deep-learning models can combine genomic, epidemiological, and spatiotemporal data to report source probabilities instead of binary cluster assignments. A U.S. CDC random forest trained on 18,661 *Salmonella* isolates achieved 81% out-of-bag accuracy when every prediction was counted and 91% when limited to high-confidence isolates whose largest predicted source probability was above 0.50. Feature-attribution analysis ranked plasmid-borne and cell-surface genes among the strongest predictors, raising host-adaptation hypotheses that still require experimental testing. The review ends with the threshold-vs.-probability debate, barriers to data sharing and federated-learning alternatives, and the gap between retrospective accuracy and prospective validation in routine surveillance.

## 1 Introduction

Foodborne diseases affect approximately 600 million people and cause 420,000 deaths each year (1, 2). Early identification of the contamination source can determine whether an outbreak is contained or allowed to spread. Over the past decade, whole-genome sequencing (WGS) has reshaped surveillance, replacing pulsed-field gel electrophoresis with cgMLST and cgSNP methods that distinguish strains differing by a single nucleotide (3–7). These methods now form the backbone of PulseNet International and of national surveillance networks worldwide.

Even these methods have hit a resolution ceiling. In clonal groups that have spread worldwide, including *S. Enteritidis* ST11, *E. coli* ST131, and *Listeria monocytogenes* ST1, the core genome is so conserved that unrelated strains differ by only a few alleles or SNPs, fewer than the usual outbreak cutoffs of seven alleles for Listeria and 10 for *Salmonella* (8–10). The result is large, spurious clusters that overwhelm investigators with non-outbreak isolates. The reason lies in how bacterial genomes evolve: the core genome records long-term phylogeny, whereas foodborne transmission and niche adaptation are driven by horizontal gene transfer in the accessory genome, which changes orders of magnitude faster (11).

These limits have pushed the field toward new methods. Pan-genomics treats accessory-gene presence or absence and mobile-element carriage as high-resolution markers, and machine learning converts those markers, together with epidemiological and spatiotemporal metadata, into probabilistic source predictions instead of binary similarity calls (12–14). We focus on the computational and statistical frameworks behind these methods rather than on wet-lab validation.

For transparency, we briefly describe how the literature was identified. We conducted a targeted, non-systematic search of PubMed and Web of Science on August 15, 2026, for English-language, peer-reviewed articles published from January 2015 through August 2026. Boolean strings combined “whole-genome sequencing,” “cgMLST,” “cgSNP,” “pan-genome,” and “mobile genetic elements” with “source attribution,” “source tracing,” “foodborne disease,” “antimicrobial resistance,” and “machine learning.” We retained original studies, methodological papers, and reviews that applied WGS, pan-genomics, or machine learning to surveillance, source attribution, or antimicrobial-resistance prediction in bacterial foodborne pathogens. We excluded conference abstracts, non-English publications, work on non-bacterial organisms, and articles in which genomic methods were not linked to inference about source, resistance, or virulence. Titles and abstracts were screened for relevance, potentially eligible full texts were reviewed, and the reference lists of key articles were hand-searched for additional studies. As this is a narrative mini review rather than a systematic review, no protocol was registered, the search was not intended to be exhaustive, and formal dual-reviewer screening and risk-of-bias assessment were not performed.

## 2 Core-genome source tracing and the threshold dilemma

### 2.1 Current methods and their limitations

Routine surveillance relies on three WGS-based approaches, whose strengths and weaknesses, particularly the trade-off between resolution and standardization, are summarized in Table 1. cgMLST compares allelic profiles across 1,500–4,000 core genes and is the most standardized of the three, with established schemes for *L. monocytogenes, Salmonella*, and Campylobacter (15–17). wgMLST extends the comparison beyond 10,000 loci to include accessory genes, sharpening resolution but increasing sensitivity to assembly quality (18). cgSNP calls core-genome nucleotide variants and builds phylogenetic trees. It offers the finest resolution for outbreak work but depends on complex bioinformatic pipelines that complicate interlaboratory standardization (19).

**Table 1:** Comparison of whole-genome sequencing-based typing methods for foodborne pathogen source tracing.

Feature	cgMLST	wgMLST	cgSNP
Genomic scope	Core genes (1,500–4,000 loci)	Core + accessory genes (>10,000 loci)	Core-genome nucleotide variants
Resolution	High; sufficient for most outbreaks	Higher; fine-scale local clusters	Highest; single-nucleotide discrimination
Standardization	High; international nomenclature servers	Moderate; sensitive to assembly quality	Low; workflow-dependent pipelines
Computational demand	Low; allele calling and distance matrices	Moderate; larger loci sets	High; mapping, variant calling, phylogenetics
Primary application	Routine surveillance and cluster detection	Local outbreak refinement	Major outbreak investigation and transmission inference
Key limitation	Fixed thresholds fail for clonal groups	Accessory loci complicate inter-lab comparison	Poor portability across laboratories

All three methods depend on fixed allele or SNP-difference thresholds to define clusters. These cutoffs are set retrospectively, at seven or fewer alleles for *L. monocytogenes* and about 10 for *Salmonella* (8, 10), and they work well for ordinary point-source outbreaks but fail in three predictable situations. First, evolutionary rates differ among pathogens and clonal groups. *S. Typhimurium* gains 1–2 SNPs per year, whereas *S. Enteritidis* changes much more slowly, so a single cutoff produces both false positives and false negatives (20). Second, sampling bias distorts cluster boundaries. A sparse background makes unrelated strains appear linked, while dense sampling reveals substructure that a threshold hides. In German Listeria surveillance, cgMLST clusters could be subdivided further by SNP methods, with prophage insertions driving the finer structure (21). Third, *L. monocytogenes* strains that persist in processing plants may show almost no core-genome change over a decade yet carry distinct prophage profiles and plasmids associated with particular microenvironments (22). In each case, the core genome lacks the variation needed to separate transmission chains. These cutoffs are operational heuristics, not universal biological constants. The performance of a given threshold depends on the pathogen's population structure and evolutionary rate, the density and representativeness of background sampling, and the epidemiological context in which clusters are interpreted. Consistent with current WGS-surveillance practice, genomic distances should be evaluated alongside epidemiological, food-chain, and biological metadata rather than treated as proof of linkage on their own. Thresholds should be calibrated, and periodically recalibrated, for each pathogen and, where necessary, for individual lineages as reference populations grow.

### 2.2 From similarity judgment to source attribution

Threshold clustering indicates only whether strains are genetically alike, not where a strain originated. In a complex outbreak with several possible sources, a list of related isolates still leaves investigators to identify the contamination source through interviews and traceback, a slow and labor-intensive process. A probabilistic ranking of candidate sources is needed, which requires both genetic markers that capture rapid adaptation and algorithms that combine genomic with non-genomic information.

## 3 Pan-genomics and machine learning: toward probabilistic attribution

### 3.1 Hierarchical genomic features across evolutionary timescales

The pan-genome, comprising core, accessory, and strain-specific genes, provides features at several evolutionary resolutions (23, 24). A practical hierarchy organizes these features into four layers rather than weighting every gene equally. The broadest layer records serotype, MLST sequence type, and clonal complex for evolutionary context. The next uses cgMLST allele profiles and core-genome SNPs to resolve medium-term relationships. A third layer comprises selected accessory-gene presence/absence patterns and functional gene clusters associated with niche adaptation. The most recent layer tracks mobile-element carriage, including plasmid replicon types, prophage families, and antimicrobial-resistance and virulence genes, to capture the latest horizontal gene transfer. Figure 1 diagrams this hierarchy.

**Figure 1:**
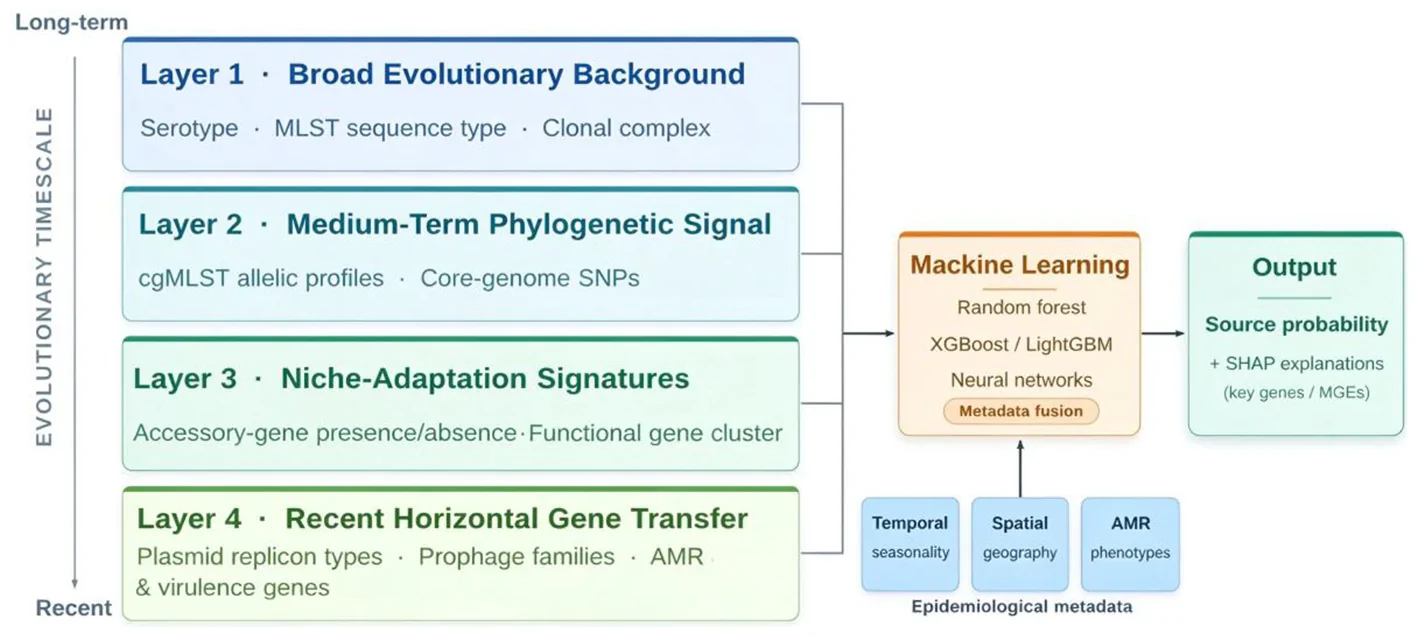
Hierarchical pan-genome feature-engineering framework for source attribution of foodborne pathogens. Four layers of genomic features span evolutionary timescales, from long-term phylogenetic background (Layer 1: serotype, MLST, clonal complex) to recent horizontal gene transfer (Layer 4: plasmids, prophages, resistance genes). Each layer is combined with epidemiological metadata (temporal, spatial, antimicrobial resistance) and fed into machine-learning models (random forest, gradient boosting, neural networks) that report source probabilities with SHAP explanations identifying the key genes or mobile elements behind each prediction.

Most of the gain in resolution comes from the accessory and mobile-element layers. Prophages can account for 10%−20% of a bacterial genome, and because they integrate at specific sites and are rarely excised, they serve as stable markers for tracing transmission chains (25). A machine-learning analysis of 24 historical *Salmonella* outbreaks found that accessory-gene content and mobile-element polymorphisms provide discriminatory signal precisely where core-genome variation is insufficient, with the most informative features enriched in membrane-transport and carbohydrate-metabolism genes (26). Plasmids carrying resistance or stress-tolerance genes likewise show strong niche specificity.

### 3.2 Machine learning and multimodal data fusion

Machine learning maps high-dimensional genomic features onto source probabilities, and Table 2 compares representative studies. Early applications of random forests, support vector machines, and gradient boosting to cgMLST or k-mer features consistently outperformed classic population-genetics methods (27, 28). In a U.S. CDC study, a random forest was trained on whole-genome MLST (wgMLST) data from 18,661 *Salmonella* isolates, each collected from a single food source, and permutation importance selected 7,360 loci, 2,987 of them core. Counting every prediction, overall out-of-bag accuracy was 0.81 (75–25 train-test accuracy 0.74; 0.77; AUC-ROC 0.93 under uniform and 0.97 under a priori class distributions), with out-of-bag accuracy of 0.95 for chicken, 0.88 for turkey, 0.83 for pork, 0.82 for vegetables, and 0.77 for beef. When the analysis was restricted to the 14,888 isolates whose largest predicted source probability was above 0.50, overall out-of-bag accuracy rose to 0.91 and chicken accuracy to 0.97. Applied to 6,470 human isolates under the 0.50 rule, the model labeled 44% as unknown or non-food; among the remainder, an estimated 46% were attributed to chicken and 27% to vegetables (29). Subsequent studies applied similar approaches to *S. Typhimurium* in the United States and Denmark, with random-forest or boosting accuracy ranging from roughly 0.78 to 0.93 depending on the algorithm, source composition, and confidence threshold (30, 31). Gradient-boosting models such as XGBoost and LightGBM generally offer the best balance of accuracy, training speed, and interpretability for public health deployment, although convolutional and graph neural networks are being explored for raw sequence and graph-structured data (32, 33).

**Table 2:** Machine-learning studies of whole-genome source attribution for foodborne pathogens, ordered by year of publication.

References	Isolates (n)	Genomic features	Algorithm	Source categories	Validation approach	Accuracy/F1/AUC	Explainability method	Major limitations
*S. Typhimurium;* Zhang et al. (30)	US livestock isolates; 8 major zoonotic outbreaks (1998–2013)	WGS; 50 selected genomic features (SNPs and accessory genes)	Random forest	Major livestock reservoirs (cattle, swine, poultry)	Retrospective outbreak attribution	7/8 outbreaks correctly attributed (87.5%)	Feature importance (top 50 features)	Retrospective only; assumes steady divergence; small outbreak set
*S. Typhimurium;* Munck et al. (31)	210 source isolates (pigs 159, broilers 34, ducks 11, layers 4, cattle 2)	cgMLST allelic profiles	Random forest; logit boost (best)	Pigs, broilers, ducks, layers, cattle (domestic/imported)	Independent validation with 95% CI	RF 0.783; logit boost 0.933 (validation 0.92); 81% of human cases predicted	Not explicitly reported	Strong class imbalance (cattle *n* = 2); 18% of cases unpredicted; single country
*C. jejuni*; Arning et al. (14)	Source genomes from chicken, cattle, sheep, wild birds, environment (environment *n* = 35)	MLST; cgMLST (1,343 genes); k-mer WGS (100,000 k-mers)	aiSource (XGBoost, best); RF, CNN, RNN/LSTM, KNN tested	Chicken, cattle, sheep, wild birds, environment	75/25 split; 200 × undersampling; balanced/unbalanced; vs. iSource	cgMLST 85%; k-mer WGS 78%; MLST 71%; iSource 64%	Bayesian human-affinity inference; confusion matrices	Host-switching generalists; sheep/cattle overlap; k-mer truncation; imbalance
*S. Enteritidis;* Bayliss et al. (34)	2,313 genomes (UK Health Security Agency)	Unitig presence/absence (25,000 selected)	Hierarchical random forest (16 node classifiers); oversampling; TPOT optimization	Geographic: 4 regions, 11 subregions, 38 countries	Held-out test; hierarchical F1; vs. flat classifier	Macro F1 0.946 region/0.724 subregion/0.670 country; micro F1 0.914; <4 min/sample	Random-forest feature selection and importance	Class imbalance; uneven global coverage; travel-volume bias
*Salmonella* (multi serotype); Rose et al. (29)	18,661 source isolates; 6,470 human isolates	wgMLST; 7,360 selected loci (2,987 core)	Random forest (1,000 trees; inverse class weighting)	15 food commodities	Out-of-bag; 75/25 train–test split	OOB 0.81 all predictions; 0.91 high-confidence (>0.50); chicken 0.97; AUC 0.93/0.97; κ 0.77	Permutation importance	Imbalanced and US-centric; cecal-isolate proxy; weak minority sources; 44% human unknown

Performance values are taken from the original reports and shown with the confidence conditions under which they were obtained.

CNN, convolutional neural network; KNN, k-nearest neighbors; LSTM, long short-term memory; OOB, out-of-bag; RNN, recurrent neural network; TPOT, tree-based pipeline optimization tool.

The main advantage over threshold methods is the ability to combine different data types. Source is associated with genetics but also with seasonality, geography, food-distribution networks, and antimicrobial-resistance profiles. Temporal features capture seasonal epidemics, spatial features reflect regional food-supply structures and can be encoded at several geographic scales, and resistance phenotypes carry source signal because antibiotic selection pressure varies among animal reservoirs. For example, a hierarchical model trained on 2,313 *S. Enteritidis* genomes from the UK Health Security Agency predicted geographic origin across four continents, 11 subregions, and 38 countries directly from WGS. It reached macro F1 scores of 0.946 at the regional level, 0.724 at the subregional level, and 0.670 at the country level (overall micro F1 0.914), with each prediction returned in under 4 min (34). Together these studies indicate that geographic structure, whether captured as travel history, regional distribution patterns, or isolate provenance, can be learned alongside genomic features to narrow the source of both travel-associated and domestically acquired infections. Tree-based models integrate these mixed data types without feature scaling and detect interactions automatically, such as that between a given prophage profile and a given region. This matters in diffuse, multi-state outbreaks where no single food vehicle is obvious and genomic, temporal, and supply-chain evidence must be weighed together.

### 3.3 Explainable AI: interpreting predictions and generating biological hypotheses

Interpretability matters when predictions guide control decisions. SHapley Additive exPlanations SHapley Additive exPlanations (SHAP) and Local Interpretable Model-agnostic Explanations (LIME) provide global feature rankings as well as explanations for individual isolates (35, 36). In *S. Typhimurium* source-attribution models, feature attribution showed that 40 of the top 50 predictive features were accessory genes carried on plasmids or prophages, and that the two leading features were non-synonymous SNPs in cell-surface genes, one in fliC, which encodes flagellin and determines serotype (30). Such findings support auditing and can highlight patterns consistent with known host adaptation and environmental survival, but one distinction must be kept clear. SHAP and LIME quantify how features affect a given model's output and therefore describe that model's behavior; by themselves they do not establish cause or mechanism. The values may reflect population structure, uneven sampling, or linkage among co-inherited genes as much as genuine biological effects, and mechanistic claims require independent experimental validation. A functionally uncharacterized gene flagged as highly predictive of source is therefore best treated as a hypothesis and a candidate for testing, feeding a loop of computational prediction, functional verification, and model refinement rather than a direct claim of mechanism (30). Local explanations remain useful during an investigation. For a single outbreak isolate, SHAP might indicate that a “poultry, 82%” prediction rests mainly on one plasmid replicon and one prophage family, giving investigators concrete feature-level leads instead of an unexplained score.

## 4 Discussion: controversies, gaps, and future directions

### 4.1 The threshold-vs.-probability controversy

Fixed thresholds appeal to traditional public health laboratories because they are transparent, reproducible, and simple to communicate: seven alleles is seven alleles in any jurisdiction and for any analyst. Probabilistic machine-learning models instead return source probabilities that vary with the training data, the algorithm, and the feature representation, and their internal logic can be difficult for non-specialists to audit. Critics argue that replacing a clear cluster definition with a probability of poultry origin introduces opacity into outbreak response and may complicate legal or regulatory action, where evidence must withstand challenge. Others respond that thresholds already convey false certainty, lumping unrelated ST11 strains together while missing real outbreaks in divergent clones, and that a probability paired with feature-level explanation is more honest than a cutoff that ignores evolutionary-rate differences and sampling context (7, 37). On balance, a layered approach is most defensible: thresholds for routine, automated screening, and machine learning for probabilistic guidance and anomaly detection where thresholds fail. This preserves the operational simplicity that surveillance laboratories require while providing higher-resolution evidence when it is needed. In either layer, genomic evidence should be interpreted alongside epidemiological and biological metadata rather than used as a stand-alone criterion.

### 4.2 Data sharing vs. commercial confidentiality

Large, representative, labeled datasets are essential, but food-industry genomic data are commercially sensitive, and even public health agencies face barriers to cross-border sharing. Public databases are dominated by clinical isolates; food and environmental isolates are underrepresented and poorly annotated, which biases predictions toward well-sampled sources and makes under-sampled reservoirs harder to detect. Federated learning offers a technical route in which institutions train models locally and share only encrypted parameter updates, never raw data, enabling collaborative model building while preserving commercial confidentiality and data sovereignty (38). In such a framework, a poultry producer, a diagnostic laboratory, and a public health agency could each contribute updates from private data without revealing strain identities, production details, or patient information. Differential privacy and secure multiparty computation add further safeguards, although they can reduce accuracy and impose computational overhead. Sustainable progress will require institutional incentives and legal frameworks that define liability and reward data contribution, not just new technology.

### 4.3 The prospective-validation gap

Prospective validation is the most consequential gap. Nearly all published source-attribution models are trained and tested retrospectively on curated datasets, and few have been integrated into routine surveillance or evaluated for their effect on outbreak-detection timeliness, decision quality, or cost-effectiveness. Retrospective accuracy does not guarantee performance under field conditions, where sampling is incomplete, bacterial populations evolve, metadata are delayed, and food systems are non-stationary. A model with 91% cross-validated accuracy can degrade as new clones emerge or food-supply patterns shift. Future studies should report alert-to-action latency, false-alarm burden, and calibration across source subgroups in addition to AUC and accuracy, and ultimately demonstrate measurable changes in outbreak outcomes. Implementation studies should compare model-augmented surveillance with standard practice in stepped-wedge or cluster-randomized designs. Closing this gap between *in silico* performance and field utility is central to wider adoption.

### 4.4 Integration with antimicrobial-resistance prediction and One Health surveillance

The same WGS and machine-learning infrastructure used for source attribution can simultaneously predict antimicrobial resistance, and resistance features themselves carry reservoir signal. Models trained on WGS, k-mer, or gene-presence data predict resistance phenotypes across many pathogens. For instance, random-forest and BioWeka classifiers applied to *Pseudomonas aeruginosa* WGS reported mean accuracy above 96% across 12 antibiotic classes while recovering known resistance determinants (39), and broader reviews document the same convergence of AI/ML with resistance prediction across pathogens and data types (40). WGS also provides an integrated view of resistance and virulence at population scale. Regional genomic surveys, including the study of *Burkholderia pseudomallei* clinical strains in Guangdong, combined MLST, SNP typing, resistance genes, and more than 160 virulence factors to characterize local strain diversity and inform surveillance (41). Embedding these capabilities in a One Health framework linking human, animal, plant, and environmental sectors is particularly relevant to food-chain surveillance, because resistant bacteria and resistance genes circulate through agroecosystems and along the food chain. Coordinated detection, mitigation, and stewardship across sectors can improve source attribution while constraining resistance (42). Delivering source and resistance prediction through shared, interoperable systems would increase the value of WGS networks such as PulseNet and strengthen the rationale for representative, cross-sector sampling.

### 4.5 Emerging technologies and conclusion

Metagenomic sequencing enables culture-independent detection directly from food and environmental samples, shortening turnaround time and capturing unculturable organisms, while single-microbe genomics can resolve mixed infections and multistrain contamination by sequencing individual cells (43, 44). Physics-guided and domain-informed methods that incorporate first-principles knowledge into learned feature spaces are also emerging. A physics-guided framework that transformed breathomic features into disease-specific representations classified respiratory diseases reliably despite sensor noise, drift, and humidity interference (45), suggesting that explicit physical or biological constraints can make learned representations more robust and transferable, a principle relevant to genomic feature engineering. Large language models may extract structured information from unstructured epidemiological reports, food-production records, and scientific literature, integrate it into source-tracing pipelines, and serve as decision-support interfaces (46).

Foodborne disease source tracing is moving from threshold-based core-genome clustering toward probabilistic attribution built on pan-genomics and machine learning. The accessory genome captures evolutionary signal missed by the core genome, machine learning converts that signal into actionable source probabilities, and explainable AI identifies the features driving each prediction and generates testable biological hypotheses rather than proving mechanisms. Realizing this potential will require layered use of thresholds and models to address the threshold-vs.-probability debate, federated approaches to overcome data-sharing barriers, and, most importantly, rigorous prospective validation of models in routine surveillance.
